# Effects of high-flow nasal cannula in patients with persistent hypercapnia after an acute COPD exacerbation: a prospective pilot study

**DOI:** 10.1186/s12890-020-1048-7

**Published:** 2020-01-13

**Authors:** Lara Pisani, Sara Betti, Carlotta Biglia, Luca Fasano, Vito Catalanotti, Irene Prediletto, Vittoria Comellini, Letizia Bacchi-Reggiani, Stefano Nava FERS

**Affiliations:** 1 Respiratory and Critical Care Unit, University Hospital St. Orsola-Malpighi, Via G. Massarenti 9, Pavilion 15, Bologna, Italy; 2 Respiratory and Critical Care Unit, Department of Clinical, Integrated and Experimental Medicine (DIMES), University Hospital St. Orsola-Malpighi, Bologna, Italy; 3 School of Medicine, Alma Mater Studiorum University of Bologna, Bologna, Italy; 4 Department of Experimental, Diagnostic and Specialty Medicine (DIMES), S. Orsola-Malpighi Hospital, University of Bologna, Bologna, Italy

**Keywords:** Chronic obstructive pulmonary disease, High flow nasal cannula, Persistent hypercapnia, Long term non invasive ventilation

## Abstract

**Background:**

Persistent hypercapnia after COPD exacerbation is associated with excess mortality and early rehospitalization. High Flow Nasal cannula (HFNC), may be theoretically an alternative to long-term noninvasive ventilation (NIV), since physiological studies have shown a reduction in PaCO2 level after few hours of treatment.

In this clinical study we assessed the acceptability of HFNC and its effectiveness in reducing the level of PaCO_2_ in patients recovering from an Acute Hypercapnic Respiratory Failure (AHRF) episode. We also hypothesized that the response in CO_2_ clearance is dependent on baseline level of hypercapnia**.**

**Methods:**

Fifty COPD patients recovering from an acute exacerbation and with persistent hypercapnia, despite having attained a stable pH (i.e. pH > 7,35 and PaCO_2_ > 45 mmHg on 3 consecutive measurements), were enrolled and treated with HFNC for at least 8 h/day and during the nighttime

**Results:**

HFNC was well tolerated with a global tolerance score of 4.0 ± 0.9. When patients were separated into groups with or without COPD/OSA overlap syndrome, the “pure” COPD patients showed a statistically significant response in terms of PaCO_2_ decrease (*p* = 0.044). In addition, the subset of patients with a lower pH at enrolment were those who responded best in terms of CO_2_ clearance (score test for trend of odds, *p* = 0.0038).

**Conclusions:**

HFNC is able to significantly decrease the level of PaCO_2_ after 72 h only in “pure” COPD patients, recovering from AHRF. No effects in terms of CO2 reduction were found in those with overlap syndrome. The present findings will help guide selection of the best target population and allow a sample size calculation for future long-term randomized control trials of HFNC vs NIV.

**Trial registration:**

This study is registered with www. clinicaltrials.gov with identifier number NCT03759457.

## Background

A number of observational studies have reported increased mortality associated with chronic hypercapnia in patients with Chronic Obstructive Pulmonary Disease (COPD) [1, 2]. For this reason, long-term noninvasive ventilation (NIV) has been suggested as a strategy to decrease the PaCO_2_ levels, with a few investigations demonstrating an improvement in survival and/or exacerbation rates [3–5].

High levels of inspiratory pressure are usually applied in an attempt to maximally reduce PaCO_2_ [4, 5], but this approach may be not well tolerated in all the patients.

An alternative method to reduce hypercapnia is the use of high flow nasal cannula (HFNC). Physiological short term studies have shown that HFNC can generate an acute reduction of PaCO2 [6–8], even over a wide variation of different baseline PaCO_2_ levels (from as low as 1.3 mmHg for a PaCO_2_ of 38.4 mmHg (− 3%) to 6.4 mmHg for a PaCO_2_ of 61.2 mmHg (− 9.2%). When compared to NIV, HFNC also demonstrated a similar reduction of inspiratory muscle effort, compared to spontaneous breathing [7].

HFNC therapy is reported to improve patient comfort [9–12], avoid mucosal dryness and injury [13–15], and deliver a more reliable and stable fraction of inspired oxygen (FiO_2_) [13, 16].

One investigation with 11 COPD patients [17] treated for 6 weeks of home HFNC, showed a significant and impressive reduction in PaCO2 compared to baseline (> 8 mmHg). However, this study had a small sample size, relied on the recording of arterialized PaCO_2_ measurements, and used a relatively low fixed flow of 20 L/min, that has been shown to have a lower CO_2_ clearance than studies using higher flow rates [17].

A randomized cross-over study in stable hypercapnic COPD patients on long-term oxygen therapy (LTOT) demonstrated that the addition of HFNC was able to improve health-related Quality of Life (QoL) and reduce hypercapnia in these patients [18]. However, the participants in this trial were atypical of most patients with severe COPD, since 37% were stage GOLD II and III and had comparatively low exacerbation and hospital admission rates, which may limit the clinical applicability of these findings [18]. Recently, it has been demonstrated that HFNC treatment reduced acute exacerbations, hospital admissions and symptoms in COPD patients with hypoxic failure [19]. This highlights the need for future randomised trials comparing HFNC vs NIV.

In fact, for patients with persistent hypercapnia following an acute life-threatening exacerbation of COPD, Murphy et al. [5] showed that the addition of home NIV to home oxygen therapy prolonged the time to readmission or death over a 12-month period.

In the current investigation, the goal was to assess the acceptability of HFNC and its effectiveness in reducing the level of PaCO_2_. The longer term research goal, based on previous physiological studies, is to demonstrate that the response of COPD patients with persistent hypercapnia after an acute exacerbation to HFNC is dependent on their baseline level of hypercapnia. This current study will help guide selection of the best target population and allow a sample size calculation for future long-term randomized control trials (RCTs) of HFNC vs NIV.MATERIAL and METHODSFifty COPD patients recovering from an acute exacerbation requiring hospital admission and with persistent hypercapnia, despite having attained a stable pH (i.e. pH > 7,35 and PaCO_2_ > 45 mmHg on 3 consecutive measurements), were enrolled in this interventional study. The protocol was approved by our local ethical committee and written informed consent was obtained from each patient. This study is registered with www. clinicaltrials.gov with identifier number NCT03759457.

Documented or highly suspected OSA/COPD overlap syndrome was not considered an exclusion criteria and was defined as the presence of 15 or more obstructive respiratory events per hour of sleep, when a previous full night polysomnography (PSG) was available (n.12 patients) [20] or from a positive Epworth questionnaire and a Body Mass Index (BMI) > 25 (n.11 patients) [21].

Cardiac decompensation, restrictive thoracic disorders, renal insufficiency, cancer, and neurological disease were considered exclusion criteria.

On day 1 the patients underwent a preliminary trial with HFNC to set the optimal flow, using the AIRVO 2 (Fisher & Paykel Healthcare, Auckland, New Zealand). The patients were asked to breathe while trying to keep their mouth closed at flow rates from 20 L/min up to 40 L/min for a minimum of 15 min, if tolerated, for each trial. At the end of this test, the maximum level tolerated for 15 min was chosen as the flow to be used for the experimental procedure. Temperature was set according to the patient’s tolerance starting from 31 °C, up to 37 °C, while FiO_2_ was adjusted to maintain an SaO_2_ between 92 and 94%.

Between 9 am of day 2 and 9 am of day 5 (72 h period), patients underwent HFNC for at least 8 h/day plus during the nighttime. The nurse on shift was in charge of supervising the adherence to this schedule and to record any protocol violations on a dedicated sheet. Recorded every morning at 10 am were: Arterial Blood Gases (ABGs) performed without HFNC, effective hours of HFNC and therapy tolerance, as measured through patient self -reporting using the following scale: 1. very bad, 2. bad, 3. moderate, 4. good, and 5. very good.

### Statistical analysis

Data are presented as a mean and standard deviation (SD). We used Repeated Measures Analysis of Variance to analyse the ABGs changes and the tolerance to HFNC during the trial. Repeated Measures Analysis of Variance were used to determine the difference between overlap syndrome COPD patients and “pure” COPD patients, without overlap syndrome. Post-hoc pairwise comparisons were performed with pairwise **Bonferroni’s** method. A test for linear trend of the odds was used to compare the baseline PaCO_2_ levels against the changes in PaCO_2_ after 48 h and pH values at baseline.

We considered two-sided *p* values less than 0.05 to be statistically significant. Statistical analysis was performed with Stata/Se 14.2 software (StataCorp, College Station TX, USA).

## Results

Table 1 shows the clinical characteristics of the patients. None of the patients were on a home care ventilator program, either due to having previously refused NIV or because they did not meet the enrolment criteria established in our unit (i.e. PaCO_2_ > 55 mmHg). All were naive to HFNC therapy. 12/50 patients were affected by overlap syndrome.

**Table 1:** Demographic data of all patients

	*n* = 50
Age, yrs	75,7 ± 9,3
Sex (M/F)	16/34
BMI	26 ± 6
FEV1 (% pred)	42,1 ± 17,4
LTOT (n/total)	27/50
History of mechanical ventilation (n/total)	26/50
“Acute” NIV during exacerbation (n/total)	35/50
Actual Smokers (n/total)	10/50
Ex-Smokers (n/total)	25/50
Charlson Index	6,4 ± 2,0
pH	7.41 ± 0.06
PaCO2 (mmHg)	60 ± 1.4
PaO2/FiO2	200.5 ± 8.7
HCO3- (mmol/L)	35.3 ± 1.03
Respiratory rate (b/min)	22.02 ± 0.6

Data are presented as mean ± SD

*NIV* Non invasive ventilation, *BMI* Body Mass Index, *FEV1* Forced Expiratory Volume in the 1st second, *LTOT* Long Term Oxygen Therapy

The protocol was well tolerated by all but one of the patients without major discomfort or any change in the time course of the experiment with regards to flow, temperature and FiO2. The mean flow applied was 33.5 ± 3.2 L/min. The exception was one patient found the warm temperature uncomfortable. HFNC was well tolerated with a global tolerance score of 4.0 ± 0.9. The mean usage of HFNC was 64.5 ± 10.5 h.

Overall, 70% of patients (35/50) received NIV prior to HFNC, most of these patients were “pure” COPD (25/35). Table 2 shows key patient characteristics according to blood gases during exacerbation and the time interval between treatments**.**

**Table 2:** Blood gases values during acute exacerbation and time interval between treatments

	Patients (*n* = 50)
pH	7.30 ± 0.05
PaCO2 (mmHg)	79.1 ± 13.7
Time interval between onset of the exacerbation and HFNC beginning (hours)	88.8 ± 40
Time interval between NIV treatment and the onset of HFNC (hours)	49.8 ± 15

Data are presented as mean ± SD

*NIV* Non invasive ventilation, *HFNC* High flow nasal cannula

Data are presented as mean ± SD

*NIV* Non invasive ventilation, *BMI* Body Mass Index, *FEV1* Forced Expiratory Volume in the 1st second, *LTOT* Long Term Oxygen Therapy

**Fig. 1:**
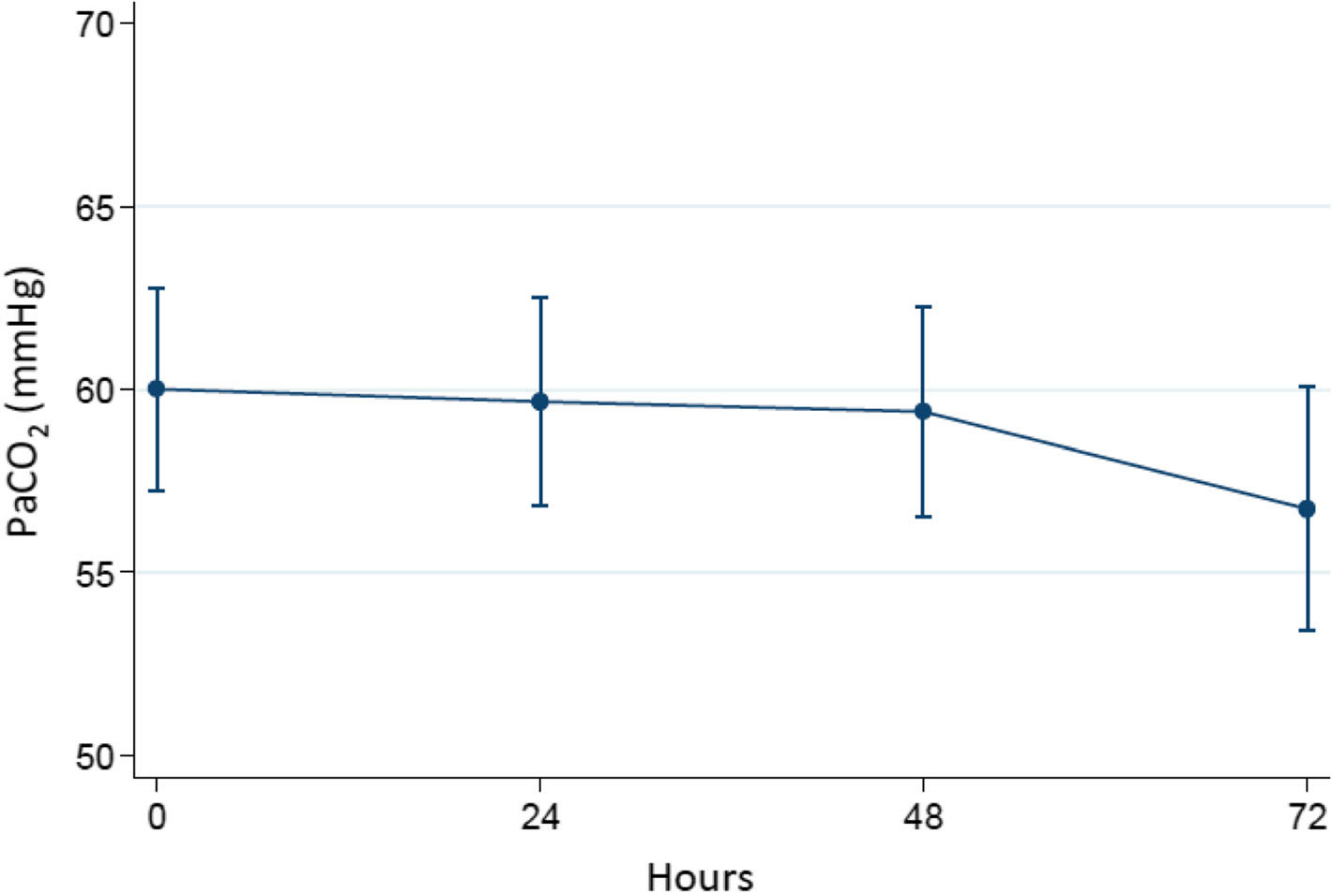
Changes in mean PaCO2 level during the trial in all patients

**Fig. 2:**
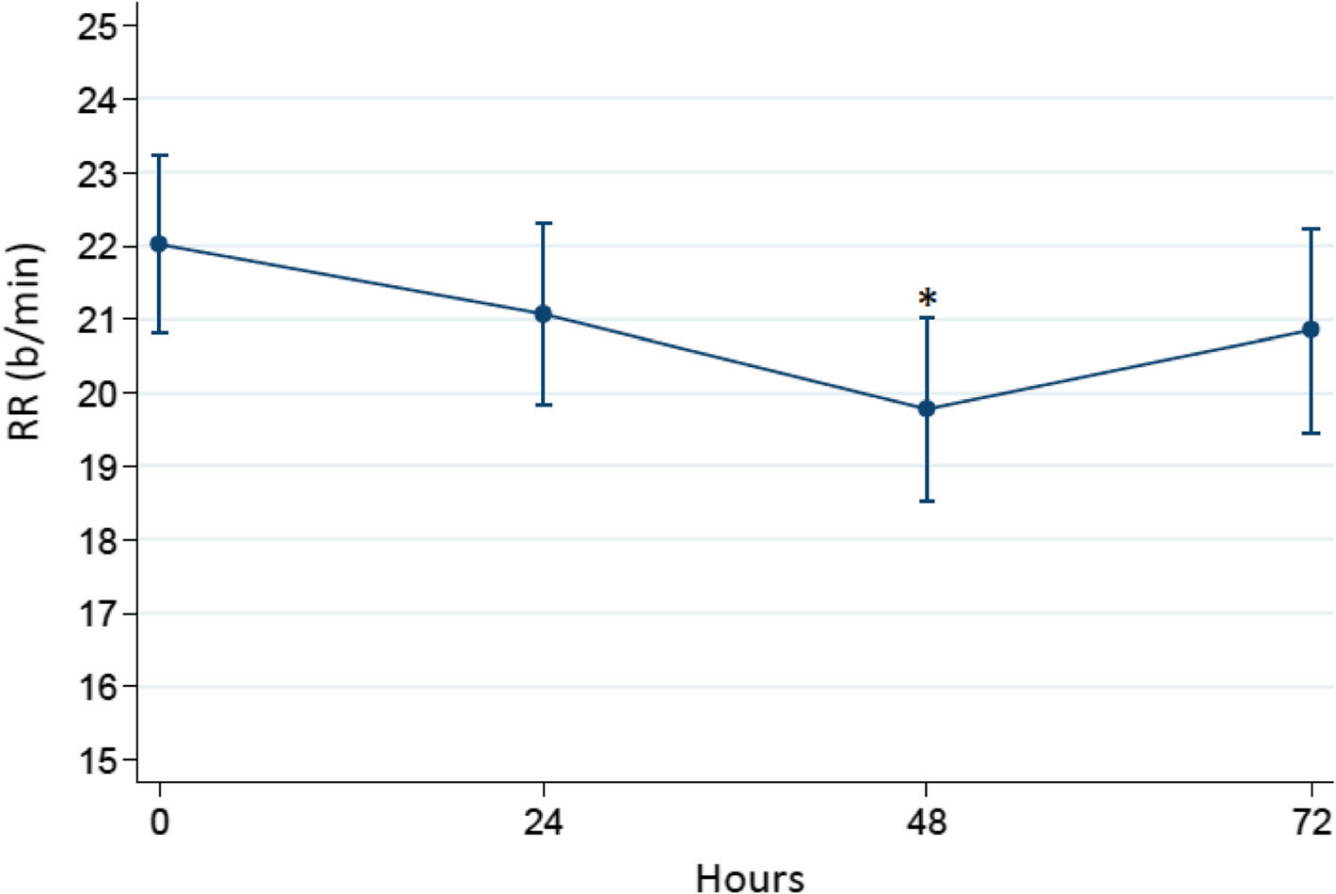
Respiratory rate during the trial

Figure 1 is a plot of the time course of PaCO_2_ during the trial. Although a positive trend of PaCO_2_ reduction was observed, no significant differences were found among the trial in all patients. However, a statistically significant reduction in respiratory rate was observed at 48 h (Fig. 2).

Table 3 summarizes the ABG values; no statistically significant differences were found in terms of pH, HCO3^−^ and PaO2/FiO2 ratio changes during the trial.

**Table 3:** ABG values during the trial

	baseline	24 h	48 h	72 h
pH	7.41 ± 0.006	7.42 ± 0.006	7.42 ± 0.006	7.43 ± 0.007
PaCO_2_ (mmHg)	60 ± 1.41	59.7 ± 1.43	59.4 ± 1.46	56.7 ± 1.7
PaO_2_/FiO_2_	200.5 ± 61	196.4 ± 58	208 ± 63	200.2 ± 66
HCO3^−^ (mmol/L)	35.3 ± 1.03	36.7 ± 1.04	36.6 ± 1.06	35.3 ± 1.2

Data are presented as mean ± SD

**Fig. 3:**
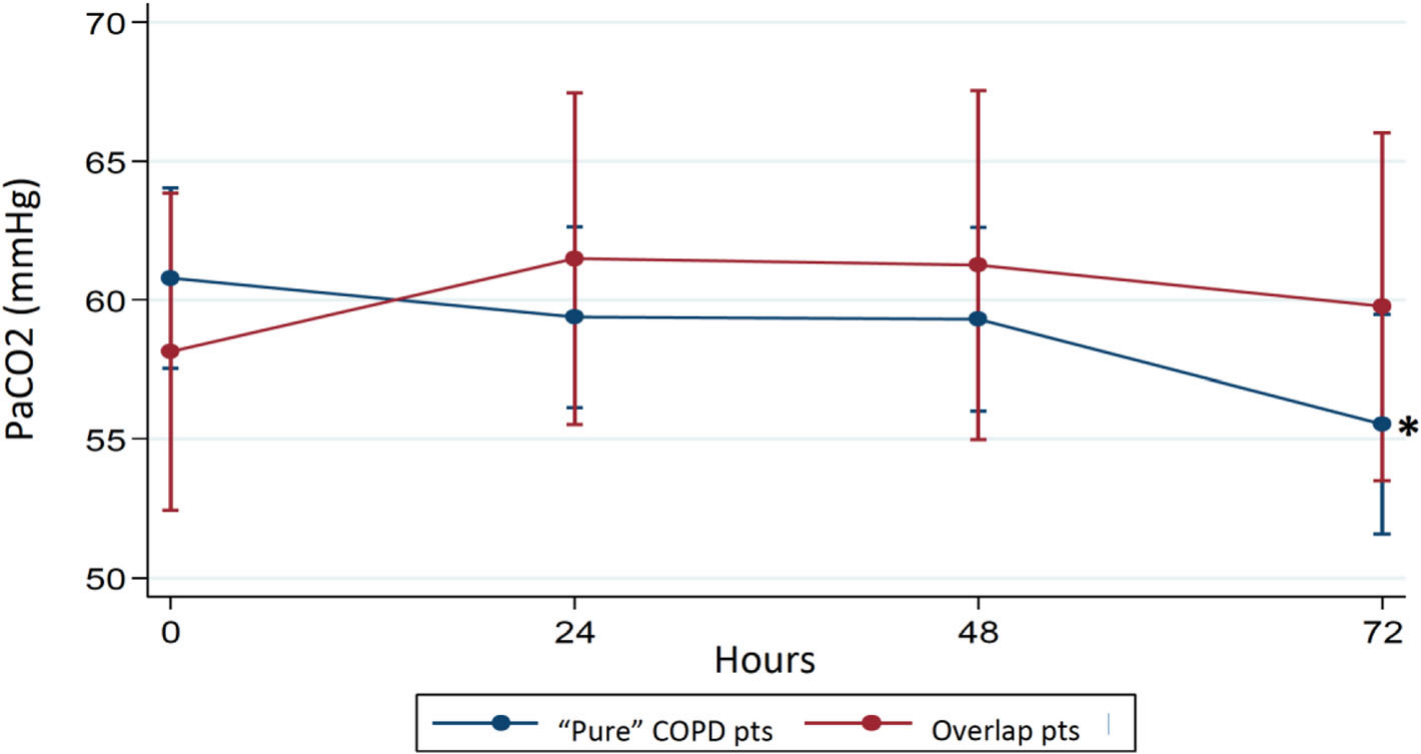
Trend of PaCO2 values during the trial according the presence or not of COPD/OSA overlap syndrome. *72 h vs baseline: *p* = 0.044(Bonferroni-adjusted *p*-values)

On the other hand, when the patients were separated into groups with or without COPD/OSA overlap syndrome, the “pure” COPD patients showed a statistically significant response in terms of PaCO_2_ decrease (*p* = 0.044). The comparison of trends of PaCO_2_ among COPD patients and subjects with overlap syndrome is shown in Fig. 3.

**Fig. 4:**
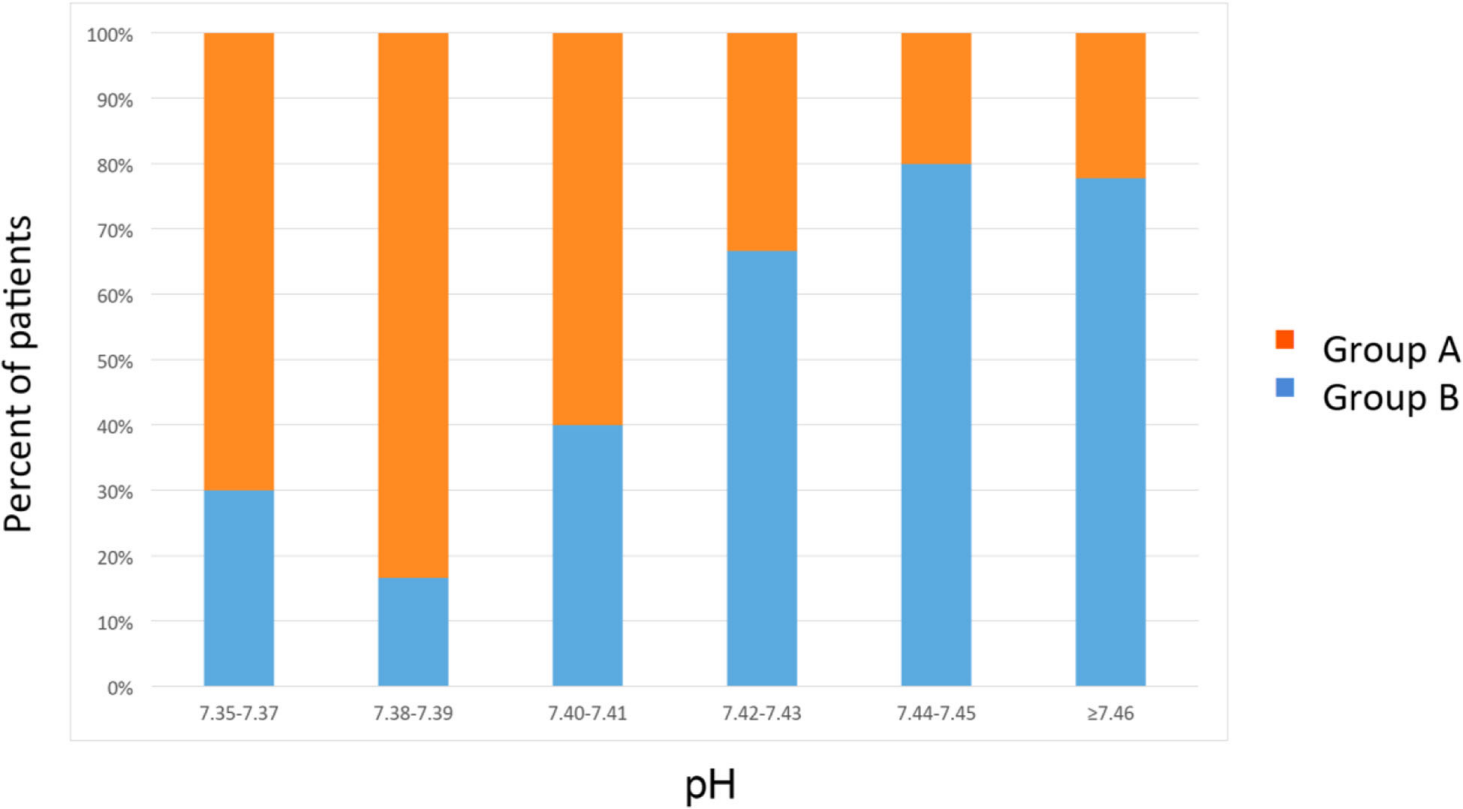
Frequency distribution of Groups A and B according to ranges of pH values. Patients were divided into two groups: those for whom a reduction of PaCO2 from baseline to 48 h occurred (Group A) and those for whom PaCO2 either increased or changed “minimally” (− 2%) (Group B). This figure shows the frequency distribution of each group according to ranges of pH values

The patients were divided into two groups: those for whom a reduction of PaCO_2_ from baseline to 48 h occurred (Group A) and those for whom PaCO_2_ either increased or changed “minimally” (− 2%) (Group B). When comparing the two groups, it was found that patients with a lower pH were more likely to respond to HFNC (Fig. 4, Table 4, score test for trend of odds, *p* = 0.0038).

**Table 4:** Score test for trend of odds between Groups A and B according to ranges of pH values

pH ranges	Group B	Group A	Odds	[95% Conf. Interval]
7.35–7.37	3	7	0.42857	0.11083 -1.65733
7.38–7.39	1	5	0.20000	0.02337 - 1.71188
7.40–7.41	4	6	0.66667	0.18813 - 2.36242
7.42–7.43	6	3	2.00000	0.50020 -7.99688
7.44–7.45	4	1	4.00000	0.44708 -35.78757
≥7.46	7	2	3.50000	0.72709 -16.84797

Score test for trend of odds: *p* = 0.0038

Patients were divided into two groups: those for whom a reduction of PaCO2 from baseline to 48 h occurred (Group A) and those for whom PaCO2 either increased or changed “minimally” (− 2%) (Group B). This table shows the Score test for trend of odds between the two groups according to ranges of pH values

## Discussion

In this study performed in patients with persistent hypercapnia after an episode of AHRF, we have shown for the first time that while HFNC is able to significantly decrease the level of PaCO_2_ after 72 h in “pure” COPD patients, this was not the case for those with overlap syndrome. In addition, the subset of patients with a lower pH at enrolment were those who responded best in terms of CO_2_ clearance. This study highlights the need for an RCT comparing the long term effects of NIV vs HFNC, in the cluster of patients more likely to respond to HFNC.

Several short-term physiological studies (a maximum of 2 h total test time) [6, 7] have shown that HFNC can improve some relevant physiological parameters, including decreasing the level of hypercapnia, in stable COPD patients.

A randomized controlled study comparing HFNC with “standard” oxygen demonstrated that the former was able to significantly reduce PaCO_2_, while improving quality of life [18].

Recently, Storgaard and colleagues [19] in a large sized study randomized 200 COPD patients with chronic hypoxemic respiratory failure to long term oxygen therapy (LTOT) or LTOT plus HFNC home treatment for 12 months. This study showed that the long-term HFNC treatment significantly reduces acute exacerbations, hospitalization and PaCO2 levels, therefore suggesting that HFNC may be an alternative to home NIV for some COPD patients [19].

As the physiological mechanisms of NIV and HFNC treatments are quite different, it is important to better understand which subset of patients are more likely to benefit from HFNC long-term application.

For example, NIV is likely to correct the mechanism leading to hypercapnia through increasing alveolar ventilation, by augmenting tidal volume while reducing respiratory rate, and reducing CO_2_ production by decreasing the work of breathing [22–24]. HFNC on the other hand may also increase tidal volume and reduce the inspiratory effort [6–8], although to a lesser extent than NIV [7], but may have additional physiological mechanisms [25].

For example, HFNC improves the lung mucociliary clearance [13–15], the washout of upper airway dead space [26–29], and generates a low level of positive airway pressure (PEEP effect) [16, 30–32], together with a decrease in inspiratory resistance and an increase in expiratory resistance [13, 33].

NIV has been suggested to decrease the number of exacerbations per year and also mortality in two different groups of COPD patients, either in a phase of clinical stability or with persistent hypercapnia after an episode of AHRF [5]. The approach used in the second patient group was probably reported to be more “accepted” and tolerated, since the drop out and non-compliant rate was about 40% in the patients enrolled when already stable vs 10% of those enrolled a few days after the hospital discharge [5].

For this reason, we decided to study the effect of HFNC in the latter cluster of patients.

We have shown for the first time that patients with a combination of COPD and sleep apnea (i.e. overlap syndrome) are not likely to benefit from HFNC treatment. Most of the previous clinical and physiological studies did not exclude a priori these subjects.

Few investigations assessed the effects of HFNC during sleep, showing that while breathing pattern response to HFNC depends on the wake/sleep state in normal healthy adults, it varies widely during wakefulness in patients with COPD [34, 35].

Indeed it was shown that during sleep these patients have a more rapid shallow breathing pattern using HFNC, compared with that of the control group on standard oxygen, but on the other hand they decreased PaCO2, as well as Work of Breathing, indicating a better alveolar ventilation [34, 35].

The authors of the latter study concluded that HFNC may be used during sleep as an alternative means to assist ventilation in patients prone to develop respiratory failure due to increased respiratory loads or insufficient alveolar ventilation [34].

Unfortunately, the patients enrolled were not likely to be affected by sleep disturbancies, as demonstrated by the low Respiratory Disturbance Index (RDI) and by the absence of relevant desaturations [34].

Despite the favourable results of the Biselli study [34], it is likely, as demonstrated in our study, that the effects of HFNC are not sufficient in overlap syndrome patients to fully relieve the increased mechanical load imposed by hyperinflation on poorly functioning respiratory muscles. NIV normalizes nocturnal hypoxemia, enhances the quality of sleep, and may even restore the hyporesponsiveness to CO_2_ [36, 37]. In addition, treatment with continuous positive airway pressure (CPAP) significantly reduces mortality and severe COPD exacerbations leading to hospitalization that have been shown to occur in these patients [38].

The present study largely confirms the data obtained with COPD patients with a pH in the normal range, whether clinically stable or recovering from an AHRF episode, showing that HFNC may be efficient in reducing PaCO_2_ and respiratory rate [6, 7, 17, 18].

In patients with hypercapnic respiratory failure, HFNC use for 6 weeks led to a decrease in capillary pCO_2_ [17] and similar results were obtained in a short term study showing not only a reduction in PaCO_2_ but also in oxygen consumption [39].

Similar changes in PaCO_2_ were described in another investigation where HFNC led to a flow-dependent reduction in PaCO_2,_ with values becoming close to normocapnia, accompanied by an increase in Tidal Volume (TV) and a decrease in minute volume, resulting in a reduction of the rapid shallow breathing index, an indicator of respiratory work load [40].

In a randomised controlled physiological crossover study, Fraser et al. [6] assessed the short-term response to HFNC therapy (30 L/min) vs conventional oxygen therapy in 30 patients. HFNC decreased transcutaneous CO_2_, inspiration to expiration ratio and respiratory rate, with a concurrent increase in End Expiratory Lung Volume and TV compared with LTOT.

In a second similar study, Pisani et al. [7] studied the effects of HFNC versus NIV on inspiratory effort in fourteen patients with hypercapnic COPD, by measuring transdiaphragmatic pressure, breathing pattern and gas exchange. HFNC and NIV were both able to significantly improve breathing pattern and reduce inspiratory effort when compared to standard oxygen; however, arterial carbon dioxide oxygen tension decreased, but not significantly [7]. Several mechanisms are involved in the explanation of these results [7, 25], such as the reduction of inspiratory resistance and anatomical dead space in the upper airways as well as the down-regulation of cold receptors or osmoreceptors in the nasal mucosa. The prolonged expiratory time may also reduce the amount of PEEPi, which may be the source of increased inspiratory load, while increasing the end-expiratory and tidal volumes, and decrease the respiratory rate [7, 25].

This study therefore highlights the potential of using HFNC as alternative to NIV in COPD patient with chronic hypercapnic patients.

The “exact” moment to start NIV in these patients is, however, still controversial. In Köhnlein’s study [4] NIV was initiated a relatively long time after an acute episode of exacerbation, but despite the favourable results, the drop out and/or compliance rate was suboptimal, while in another RCT [5], showing similar results, NIV was started after an episode of AHRF requiring NIV treatment and the compliance rate was much better overall.

As already stated, all the studies using HFNC were performed a relatively long time after an episode of AHRF, and in patients not very likely to be ideal candidates for long-term NIV, because of the level of hypercapnia, their severity stage and the number of previous exacerbations. Therefore these results should not be considered when designing a RCT, with a head-to-head comparison between NIV and HFNC in patients likely to benefit more from long term NIV.

Interestingly enough our results also showed that in this subset of patients, the greatest PaCO_2_ reduction was observed mainly in patients with lower pH, despite being in the normal range.

Our study has some limitations. First of all, not all the patients with overlap syndrome performed a full night polysomnography, but obviously their medical history, BMI and Epworth questionnaire suggested a likely combination between COPD and sleep apnea syndrome. Secondly, one may argue that the mean flow rate applied (33 L/min), may be suboptimal to obtain a maximized CO_2_ clearance [41]. However, the flow employed in this study was based on the patient’s tolerance, which is the key to success when proposing a long-term chronic application.

Finally, the study was performed in the recovery phase of acute COPD exacerbation, that means the patients might be still in the process of PaCO2 reduction, especially in the subset of patients with a lower pH at enrolment [42]. Therefore, it is difficult to attribute the CO2 reducing effect to HFNC alone without a control group. However, the hypothesis tested in this study is still legitimate as the fact that home NIV and long term oxygen therapy (LTOT) has less physiological and clinical effect in patients with the reversible hypercapnic phenotype [42, 43]. In addition, in this “proof of concept” study, we wanted to show that HFNC is able to reduce PaCO2 not only in the stable phase, as already shown, but also after an acute exacerbation, that is nowadays the “ideal” target for starting home NIV, as suggested by the recent European Respiratory Society guidelines on long-term home non-invasive ventilation [44].

## Conclusions

In conclusion we have shown that in COPD patients recovering from an episode of AHRF, which have reached a normal pH, the use of HFNC was associated with a statistically significant reduction in PaCO_2_ and respiratory rate. The better response was obtained in the subset of individuals with a lower pH level. This was not the case for COPD patients with the overlap syndrome. The results of this study may be useful to determine the sample size and the “ideal” characteristics of patients to include in an RCT aimed at assessing the efficacy of HFNC vs NIV in COPD patients recovering from an episode of AHRF.

## Data Availability

The datasets used and/or analysed during the current study are available from the corresponding author on reasonable request.

## Abbreviations

ABGs: Arterial Blood Gases
AHRF: Acute Hypercapnic Respiratory Failure
BMI: Body mass index
CO2: Carbon dioxide
COPD: Chronic Obstructive Pulmonary Disease
FiO2: Fraction of inspired oxygen
GOLD: The Global Initiative for Chronic Obstructive Lung Disease
HFNC: High Flow Nasal Cannula
LTOT: Long-term oxygen therapy
NIV: Noninvasive ventilation
OSA: Obstructive sleep apnea
PaCO2: Arterial partial pressure of carbon dioxide
PEEP: Positive end-expiratory pressure
PEEPi: Intrinsic positive end-expiratory pressure
PSG: Polysomnography
QoL: Health-related Quality of Life
RCT: Randomized controlled trial
RDI: Respiratory Disturbance Index
SaO_2_: Oxygen saturation
SD: Standard deviation
TV: Tidal Volume
